# Impact of Pediatric Mobile Game Play on Healthy Eating Behavior: Randomized Controlled Trial

**DOI:** 10.2196/15717

**Published:** 2020-11-18

**Authors:** Yi-Chin Kato-Lin, Uttara Bharath Kumar, Bhargav Sri Prakash, Bhairavi Prakash, Vasini Varadan, Sanjeeta Agnihotri, Nrutya Subramanyam, Pradeep Krishnatray, Rema Padman

**Affiliations:** 1 Hofstra University Hempstead, NY United States; 2 Center for Communication Programs Johns Hopkins University Baltimore, MD United States; 3 FriendsLearn Inc Palo Alto, CA United States; 4 The Mithra Trust Chennai India; 5 Mind in Motion Chennai India; 6 Center for Communication and Change – India New Delhi India; 7 Seethapathy Clinic & Hospital Chennai India; 8 The Heinz College of Information Systems and Public Policy Carnegie Mellon University Pittsburgh, PA United States

**Keywords:** pediatric obesity, mobile games, implicit learning, healthy eating behavior evaluation, game telemetry analysis

## Abstract

**Background:**

Video and mobile games have been shown to have a positive impact on behavior change in children. However, the potential impact of game play patterns on outcomes of interest are yet to be understood, especially for games with implicit learning components.

**Objective:**

This study investigates the immediate impact of fooya!, a pediatric dietary mobile game with implicit learning components, on food choices. It also quantifies children’s heterogeneous game play patterns using game telemetry and determines the effects of these patterns on players’ food choices.

**Methods:**

We analyzed data from a randomized controlled trial (RCT) involving 104 children, aged 10 to 11 years, randomly assigned to the treatment group (played fooya!, a dietary mobile game developed by one of the authors) or the control group (played Uno, a board game without dietary education). Children played the game for 20 minutes each in two sessions. After playing the game in each session, the children were asked to choose 2 out of 6 food items (3 healthy and 3 unhealthy choices). The number of healthy choices in both sessions was used as the major outcome. We first compared the choice and identification of healthy foods between treatment and control groups using statistical tests. Next, using game telemetry, we determined the variability in game play patterns by quantifying game play measures and modeled the process of game playing at any level across all students as a Markov chain. Finally, correlation tests and regression models were used to establish the relationship between game play measures and actual food choices.

**Results:**

We saw a significant main effect of the mobile game on number of healthy foods actually chosen (treatment 2.48, control 1.10; *P*<.001; Cohen *d*=1.25) and identified (treatment 7.3, control 6.94; *P*=.048; Cohen *d*=.25). A large variation was observed in children’s game play patterns. Children played an average of 15 game levels in 2 sessions, with a range of 2 to 23 levels. The greatest variation was noted in the proportion of scoring activities that were highly rewarded, with an average of 0.17, ranging from 0.003 to 0.98. Healthy food choice was negatively associated with the number of unhealthy food facts that children read in the game (Kendall τ=–.32, *P*=.04), even after controlling for baseline food preference.

**Conclusions:**

A mobile video game embedded with implicit learning components showed a strong positive impact on children’s food choices immediately following the game. Game telemetry captured children’s different play patterns and was associated with behavioral outcomes. These results have implications for the design and use of mobile games as an intervention to improve health behaviors, such as the display of unhealthy food facts during game play. Longitudinal RCTs are needed to assess long-term impact.

**Trial Registration:**

ClinicalTrials.gov NCT04082195; https://clinicaltrials.gov/ct2/show/NCT04082195, registered retrospectively.

## Introduction

### Background and Significance

Obesity is an increasingly common epidemic in children, with serious long-term health consequences and high health care costs [1,2]. While there are many factors that contribute to overweight and obesity, dietary decisions are a leading cause [1]. Threats to pediatric health risk from the double burden of overweight and malnutrition or undernutrition heightens the need to influence children’s dietary lifestyle habit formation through evidence-based, scalable, digital therapeutic methods [3]. There is a clear need to identify effective methods for improving dietary intake and physical activity habits early in life that children find engaging and can remain intrinsically motivated to adopt.

Several diet-related video games have been designed and evaluated for children in recent years, such as Diab [4], Squire’s Quest! [5], Squire’s Quest! II [6], Quest to Lava Mountain [7], D.W.’s Unicorn Adventure [8], Alien Health Game [9], Fitter Critters [10], Creature-101 [11], Virtual Sprouts [12], Fit, Food, Fun [13], and Garfield vs Hotdog [14]. Most of the video games being evaluated predominantly use explicit education strategies such as providing answers, feedback, instructions, or suggestions to the players [4-6,9-13]. Implicit learning is another strategy that educates players without making them aware of the fact [15]. This is a rapidly growing field due to the high popularity of video games on mobile devices in general and serious games for health and education in particular. Lumosity [16] is one such well-known game, which aims to improve behavioral and cognitive performance by stimulating players’ brain function [17]. Several studies have shown its positive effect [17,18]. Without explicitly educating the players, implicit learning embedded in video games has also been found to improve actual sports behavior and physics knowledge [19,20]. However, evidence of the effects of games with implicit education strategies on pediatric healthy eating behavior is still very limited.

Serious games are generally considered an effective intervention for influencing healthy eating behaviors in children [21,22], with some exceptions [9,12,14]. There are studies providing evidence of their positive effects on food intake [4] and healthy eating attitude and self-efficacy [10]. Hence, personalizable gamification and learnification on mobile devices may be one approach for children to learn about healthy dietary habits in a fun and enjoyable way, especially mobile games embedded with implicit learning strategies. Video games include many levels of challenges, imaginative virtual worlds, and the opportunity to navigate them in distinct ways. While much is known about the design of serious games, recent research has highlighted the gap in understanding probable links between game-playing behaviors and observed outcomes so that game design and redesign can be informed through evidence-based knowledge and practices [23-25]. Existing randomized controlled trials (RCTs) predominantly compare health-related outcomes between treatment and control groups without examining how the games are played [21,22,26]. To design appropriate interventions in the game environment for children’s health-related behavior formation and change, we need to learn more about the underlying patterns of player behaviors or engagement evidenced during gameplay.

The availability of highly granular game telemetry—data obtained about the actual clicks made by players as they navigate the game—provides a unique opportunity to understand the potential associations between game-playing behaviors and observed outcomes. These clickstream data are usually used to identify flaws in game design and improve user retention measures such as churn and attrition [27,28]. The growing field of game analytics mostly focuses on using game telemetry to discover and communicate meaningful characteristics in the context of game development and impact [29]. For example, Westera and colleagues [30] use aggregated game metrics, such as total time played, number of user actions, and number of videos viewed, to describe student engagement and associate these aggregated variables with student learning outcomes. Similarly, Sharma and colleagues [7] examine the relations between aggregate game metrics (time spent in game play and levels played) and health behavioral outcomes. However, the dynamic of game play was not captured, missing potential insights from the flow of the game play, resulting in a gap in existing studies [31]. Additionally, identifying which paths result in the longest game time and what game features engage players in these paths require methods to learn paths from detailed game telemetry and examine the features that define these paths in unique ways. Loh and Sheng [32] explore the dynamics of game play and portray players’ game play behaviors by constructing game play paths/sequences for each user and using the paths to classify game play experts from novices. However, in the current literature, limited attention has been paid to the impact of these paths, which incorporate aggregate game play data and dynamics of the game play, on health-related behavioral outcomes.

In this study, we analyze the relationship between game play patterns and dietary health–related behavioral outcomes using the mobile serious game fooya!, an action video game designed to promote healthy eating and physical activity in children [33]. The primary education components are implicitly embedded in the game, aiming to make players think deeply and strategically, without the game explicitly showing the right answers. For example, the avatar in the game collects highly prized coins by fighting against enemies made up of unhealthy foods. Building on prior descriptive work [34], we first examine the immediate impact of fooya! on children’s food choices in comparison with a game without educational components (Uno board game) using an RCT study design. We then examine the various patterns of player engagement and investigate their effects on player food choices. Observed food choice data, game telemetry, and survey data are collected from the RCT, and a relevant subset of the data are analyzed in this study.

This study adds to the literature on serious health games for children by evaluating the effect of a video game with implicit learning on dietary behavior. In addition, this study makes two novel contributions to the literature. First, while it is well known that children have different play patterns during game play, our research quantifies and measures these differences by looking into the detailed game telemetry and constructing measurable variables such as the length of the activity sequence, number of transitions between actions, and frequency of reading food facts in contrast with analyzing survey, interview, and observational data and serves as the first step for evidence-based personalization. Second, this paper provides novel and granular insights into designing more impactful interventions on mobile games using the lens of examining the complex interactions between game playing patterns and health behaviors.

### Objectives

The objectives of this study are to (1) examine the immediate impact of a pediatric dietary mobile game with implicit learning on children’s actual food choices, (2) quantify children’s heterogeneous game play patterns, and (3) understand the effects of game engagement by associating game play patterns with players’ actual food choices.

### Theoretical Background

The key knowledge for eating healthfully is implicitly embedded in fooya! (eg, the fact that the avatar’s speed and body shape vary in response to the type of food intake) and also presented as nutrition facts at the end of each level. The output of implicit learning, representing the practical knowledge needed to perform a behavior [15], is necessary for behavioral change [35]. This knowledge can be delivered effectively in a game if its engaging nature can trigger children’s intrinsic motivation to pay attention to and engage in the game-like neurocognitive training [36]. This enactive learning process—learning by doing and experiencing the consequences of one’s actions—that the players experience virtually in the game, through the actions of the avatar, also contributes to behavioral change [35,37]. As promoted by the Institute for the Learning Sciences [38], learning by doing is an approach for mastering some real-world tasks. The learning experience in the game can be further transferred to real-world principles, without instructions to concentrate on problem similarity [39,40]. This suggests that actions that result in healthier body shape are retained and those that lead to negative consequences, such as heavier body shape, will be refined or discarded in the real world, which motivates our objective 1.

While children may learn from the experience, they may learn in different ways. Learning styles are often used to describe the distinct characteristics of children during their learning process, such as their use of problem-solving strategies and decision-making behaviors [41-44]. Different learning styles are found to be related to children’s learning outcomes, such as grades, spoken language, and general conduct [41,43,45-47]. The form-function shifts model by Saxe [48,49] assumes that development of thinking can be understood through examination of children’s goal-directed activity, which is also true in game environments. For example, Buckley and Doyle [47] find several relationships between learning styles and game engagement. Hassan and colleagues [42] are able to predict students’ learning styles using play logs with 72% to 78% accuracy. Nasir [50] analyzes video tapes to understand players’ cognitive learning when playing a video game. Therefore, for objectives 2 and 3, we assume that different patterns of game play may lead to different learning outcomes.

In portraying children’s engagement in the game for objectives 2 and 3, we have adapted the MDA (mechanics, dynamics, aesthetics) framework of Hunicke and colleagues [51]. Aesthetics, which “describes the desirable emotional responses evoked in the player when she interacts with the game system” [51], was not included in our paper as it was unobserved. We define player’s reactions to incentives (labeled as incentives) and behaviors in learning about foods (labeled as food learning) as two separate components, as opposed to the paper by Hunicke and colleagues [51], which includes these two in mechanics. Therefore, in this paper, we define game mechanics as the various avatar activities and game features that were experienced by the player (excluding incentives and food learning), game dynamics as the observed responses when the players interact with game mechanics, game strategies as players’ reactions to incentives, and food learning as players’ choices in learning food-related knowledge in the game.

## Methods

### Intervention

The intervention used in this study, fooya!, is a mobile gaming app available on iOS and Android platforms that employs mechanisms of implicit learning [33]. Based on experiments and hypotheses derived from pediatric neuropsychology [52], fooya! was developed to deliver therapeutic entertainment that makes healthy behavior change fun for the children. Through prior pilot studies, fooya! has been shown to deliver positive outcomes with respect to food choices, self-reported dietary choices, and healthy eating intentions [34].

**Figure 1:**
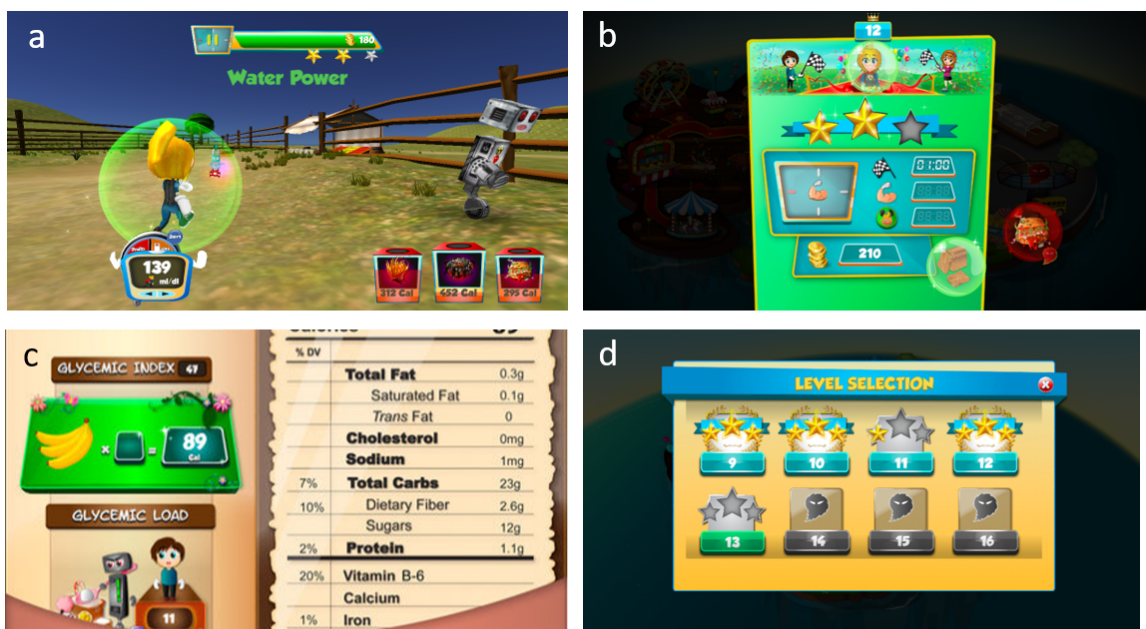
Screenshots of Fooya: (a) player is shielded by the bubble after consuming water, (b) performance summary after completing a level, (c) nutrition facts of a chosen food, and (d) level selection.

Fooya! is designed as an epic action game with an avatar fighting against robots that represent unhealthy/bad foods. Players present themselves as avatars in fooya!, which currently has 80 game levels that progress with increasing difficulty. In each level, the players’ main goals are to maintain a good body shape for the avatar and earn enough coins to win the level and unlock the next level. The body shape changes according to the avatar’s food consumption and physical activity (running/jumping) movements, and the changes affect the speed of the avatar. As the avatar consumes more calories, it becomes heavier and its movements become slower. Incentives such as highly prized coins can be earned by destroying the robots (composed of bad foods) using ammo (short for ammunition, composed of bad foods), which implicitly conveys the idea of getting rid of bad foods. Another way to earn the coins, at twice the rate, is by hitting the robots using a shield obtained by consuming good foods (Figure 1a). This feature implicitly conveys the idea that good foods can provide protection from the negative effects of unhealthy foods. After a level is finished, the screen will display a performance summary (Figure 1b) as well as all the food items encountered by the player in that level. The player can choose to click and read these nutrition facts (Figure 1c). Winning a level unlocks the next level, and the player can choose either to replay a level or proceed to the next level (Figure 1d). Throughout the game, the players were not explicitly taught what the healthy and unhealthy foods are and what foods the avatar should consume. The app has no conversation built into the game at any stage, hence explicitly teaching children about healthy/unhealthy foods using language features is not possible. It is designed to be language neutral and has no literacy requirement for use.

### Experiment and Data

An RCT with pre- and posttreatment measurements was conducted to achieve the objectives of this study. FriendsLearn Inc provided technology access, data collection and extraction, and management support [53]. Power analysis indicated that, with an effect size of 0.50, alpha level of .05, and power of 0.80, at least 102 children needed to be recruited [54,55]. The RCT was conducted in Chennai, India, and had a sample of 104 participants aged 10 to 11 years (same grade) recruited from 3 classes in 3 schools with similar socioeconomic background, one from each school. All students in the participating classes were eligible to join the study. Approvals and availability of class schedules to complete the experiment were confirmed with the school principals and grade teachers prior to the study design. Additionally, all participating students and their parents provided informed consent, and students were randomly assigned to control and treatment groups (Table 1). Demographics (gender, BMI, baseline food preference) were not significantly different between the control and treatment groups (*P*>.17). Institutional review board approvals from the Center for Media Studies in India, Carnegie Mellon University, and Hofstra University were also obtained. This RCT was retrospectively registered on ClinicalTrials.gov [NCT04082195]. No alterations were made to the experiment design after the RCT began.

**Table 1:** Sample size, random assignments, and demographic distribution.

Demographics	Treatment group schools (n=52)			Control group schools (n=52)			*P* value^a^
	A (n=20)	B (n=16)	C (n=16)	A (n=19)	B (n=17)	C (n=16)	
Male, n (%)	11 (55)	8 (50)	6 (38)	13 (68)	9 (53)	12 (75)	.63
Female, n (%)	9 (45)	8 (50)	10 (63)	6 (32)	8 (47)	4 (25)	—
BMI (kg/m^2^), mean (SD)	17.3 (3.20)	18.1 (4.13)	—^b^	19.2 (6.26)	19.2 (4.42)	—	.17
GoodBase^c^, n (%)	8 (50)	3 (23)	3 (21)	12 (86)	5 (29)	7 (50)	.29

^a^*P* values were reported from chi-square tests for gender and GoodBase and 2-sample *t* test for BMI for the pooled sample from schools A and B only, because data from school C were excluded when performing statistical tests for objectives 1 and 3.

^b^BMI for school C was not collected.

^c^GoodBase captures the number of students who reported good food as their favorite food at baseline. There are some missing values for this variable.

A structured pretest questionnaire was used to collect demographics, food eaten in the previous week, food preferences, and use/frequency of playing video games. Students in the treatment group played fooya! for 20 minutes in a continuous session during the school day, while the control group played a board game (Uno), which does not deliver any knowledge about healthy eating. After the game, the children were shown 3 pairs of food items (healthy and unhealthy) offered from 3 categories—drinks (water and Nimbooz, a carbonated soft drink), savory snacks (cashews and Lays potato chips), and sweet snacks (raisins and a 5 Star chocolate bar)—and asked to choose 2 items. These food items were chosen due to their popularity among children of that age in that region of India. Food selection was conducted in a separate room from the game playing classroom, except in school C, where students made their food choices in the game playing room with other students present. Because this posed the risk of potential contamination of their food choices, school C was excluded from all analyses related to food choices. After the food choice, students were allowed to consume the selected food items.

**Figure 2:**
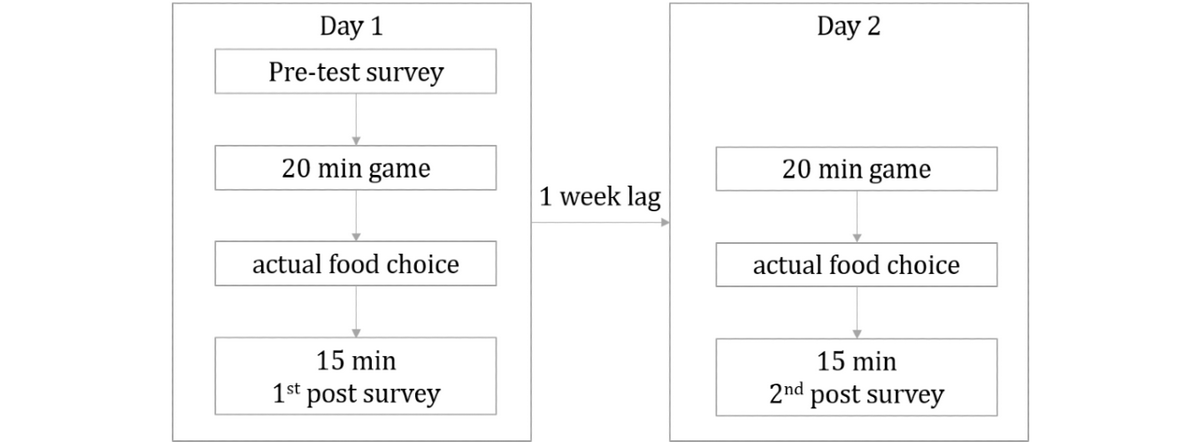
Study procedures.

Research staff who recorded the food choice responses were blinded to whether the kids were from the control or treatment group, and participants were blinded to the purpose of the study. Finally, a posttest questionnaire was administered to collect information similar to the pretest questionnaire but with additional information on nutritional knowledge. One week later, the same procedure was administered again with the exception of the pretest survey. Therefore, the children were exposed to the intervention twice, 1 week apart, each for 20 minutes, with children mostly picking up the game from where they left off in the first session. Figure 2 illustrates the study procedures.

**Figure 3:**
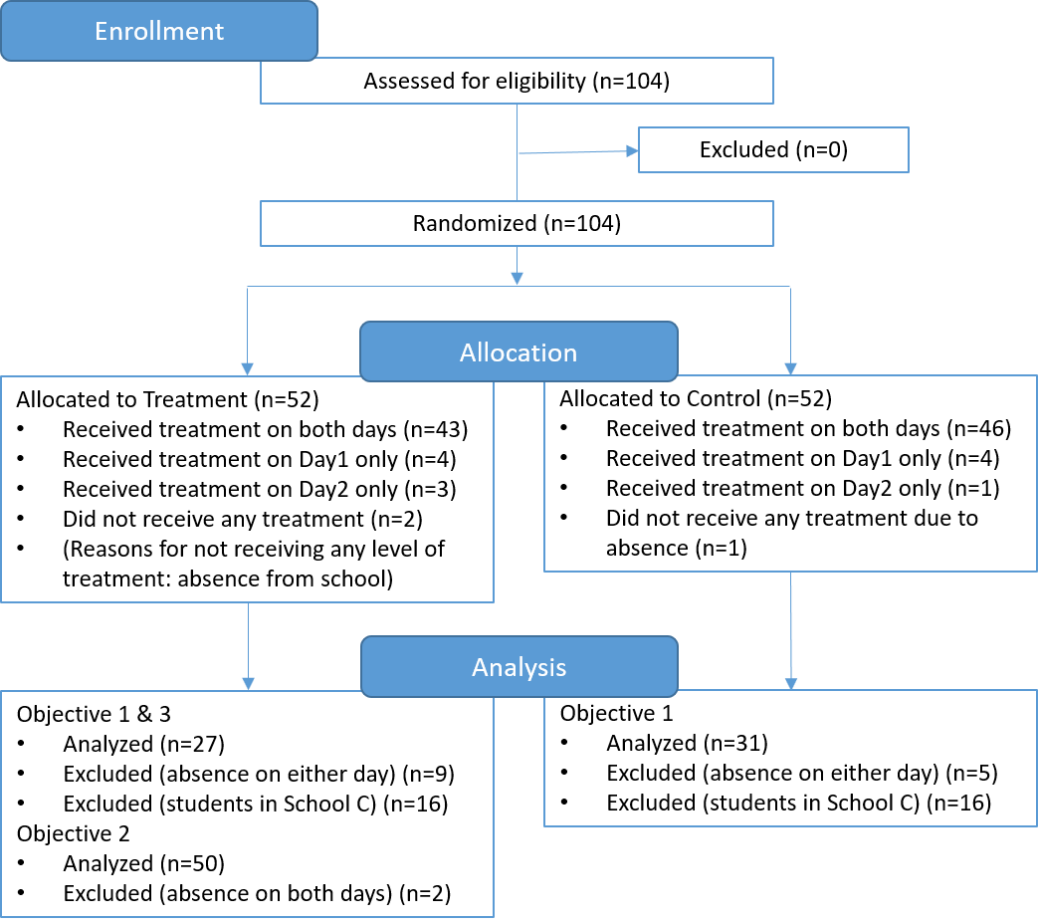
Consolidated Standards of Reporting Trials flow diagram of participants through the trial.

This study is based on a subset of the data collected during the above-mentioned RCT. We analyzed data collected from three sources: survey data (one question each from pretest and posttest), observations of the children’s actual food choices, and game telemetry that included about 65,000 game play actions/clicks. Objectives 1 and 3 were investigated using the data collected from schools A and B, and objective 2 was analyzed using the data from all three schools. Survey data that were irrelevant to our objectives were not analyzed or reported. Figure 3 provides the details of the flow of participants and data being analyzed.

### Analysis for Objective 1

To analyze the main effect on actual food choice (objective 1), the total number of good foods chosen in two exposures by the treatment and control groups was compared. We also examined the main effect on food knowledge, measured by one survey question asking the students to identify healthy food items from a list of 4 items. The total number of correctly identified healthy food items in two surveys was compared between the control and treatment groups. As secondary outcomes, the effects on the changes of both major outcomes were assessed. Mann-Whitney *U* tests were applied to avoid normality assumption. Cohen *d* was calculated to obtain effect size.

### Analysis for Objective 2

To explore children’s game play patterns (objective 2), we focused on the telemetry data generated by the participants assigned to the treatment group. Our goal was to understand how each game feature was used and summarize play patterns when children encountered the features for the first time. Therefore, only the data associated with the first encounter of a level by each child was analyzed even if children repeated playing a level a second time.

First of all, each player’s telemetry data were processed and encoded according to a feature labeling scheme by which every feature in the game was assigned a distinct feature label. Labels S and E were used to indicate the initiation and termination, respectively, of the level. Labels 1 to 5 represent players’ events in the game associated with their avatar (Table 2): (1) consuming good/healthy foods to shield themselves from bad/unhealthy food robots (good food shield), (2) destroying a robot using a shield (destroy by shield), (3) shooting bad food ammo (bad food shot), (4) destroying a robot using bad food shots (destroy by shot), and (5) being hit with bad foods thrown by robots (hit). These labeled events were ordered chronologically to form a sequence based on their recorded timestamps. For example, the time stamped events shown in Table 3 will result in a sequence of S124331E, which has a sequence length of 8 with 6 transitions. More details are described elsewhere [34], and our method of sequence labeling is consistent with existing studies [32,56,57]. These sequences were used to model the process of game playing at any level across all students as a discrete, time-homogeneous, first-order Markov chain with 7 states, one for each labeled event.

In addition to describing the game play patterns as a model, we further defined game play measures using the MDA framework [51]. The first component in the MDA model is game mechanics, measured as the highest level played in the 2 sessions (Level_max) and average sequence length across levels played (Avg_Seqlen). The second component is game dynamics, which describes how swiftly, or dynamically, the player navigates different features. This is measured by the average number of transitions to a different state in a level across all levels played (Avg_transition). The third component is game strategies, which capture how the player reacts to game rewards. Some might not react to the reward program, others may work hard to get rewards, and still others may figure out the approach to earn rewards strategically. This is measured by the proportion of robots destroyed by shield (Sum2_Sum2+4). To make sure that this is a legal fraction, we use 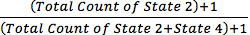 for each player. This measure is chosen to reflect the fact that destroying a robot by shield is highly rewarded with coins and thus demonstrates how strategically a player is amassing rewards. The last game component is food learning. We measure this by counting the average number of good and bad food facts read per level (AvgGFact and AvgBFact, respectively). Descriptive statistics are calculated to summarize these variables.

**Table 2:** Definitions of each feature label.

Label	Definition
S	Player starts the level
E	Player finishes the level
1	Player consumes good food to generate shield
2	Robot killed by good food shields
3	Player shoots bad food ammo
4	Robot killed by bad food shots
5	Player hit by bad food robot

**Table 3:** Example of timestamped data and labeling producing sequence S124331E.

Level	Date	Time	Status	Label/State
Level 1	7/10	09-20-19-775	Player starts the level	S
Level 1	7/10	09-20-26-482	Player consumes good food to generate shield	1
Level 1	7/10	09-20-28-956	Robot killed by good food shields	2
Level 1	7/10	09-21-10-714	Robot killed by bad food shots	4
Level 1	7/10	09-21-10-814	Player shoots bad food ammo	3
Level 1	7/10	09-21-10-894	Player shoots bad food ammo	3
Level 1	7/10	09-21-14-717	Player consumes good food to generate shield	1
Level 1	7/10	09-21-20-281	Player finishes the level	E

### Analysis for Objective 3

To examine the effects of player engagement on actual food choices (objective 3), we first identified the game play measures (the 6 measures for the MDA framework defined in objective 2) that were associated with the behavioral outcome. To this end, we conducted Kendall τ correlation tests (to avoid the linearity assumption) and stepwise feature selection using the step() function in the stats package in the R software (R Foundation for Statistical Computing). The identified variables were then included as explanatory variables in regression models for the total number of good foods chosen in 2 sessions (GoodChoice, ranging from 0 to 4) [36]. We considered modeling GoodChoice as normal and Poisson distributions. The normal distribution was chosen as it had the lowest Akaike information criterion for the distribution fit (values for normal and Poisson were 79.64 and 84.23, respectively). Given the fact that GoodChoice was a count measure, a Poisson regression was also run. We used the data pooled from the 2 sessions to avoid spillover effects. Similar to objective 2, only the first-encounter sequences were used in this analysis.

## Results

### Objective 1

For objective 1, we found strong evidence of the positive main effect of the mobile game on food choices (Table 4). Specifically, children from the treatment group chose 1.38 more good foods during the 2 exposures, on average, than the control group (treatment 2.48, control 1.10; *P*<.001; Cohen *d*=1.25). The power to detect such an effect size, with an alpha level of .05 and sample size of 58, was more than 99% [54,55]. The number of healthy foods correctly identified by the treatment group in the posttest survey question was also significantly higher than the control group (treatment 7.3, control 6.94; *P*=.048; Cohen *d*=.25).

When comparing the change in both outcomes (day 2 – day 1) between the two groups, no statistically significant results were found, suggesting that the impact of the intervention on healthy food choices after the first session may be sustained after the second session. Additionally, the nonsignificant effects of the game on the change in food choices may have been partly due to the low dosage the children received in the experiment—only 40 minutes in two sessions combined, played 1 week apart. This result supports both the theory and literature, described in the Introduction, that game play experience is associated with health behavioral outcomes. Next, we investigated the potential patterns during game play that were related to this observed positive main effect with analyses of objectives 2 and 3.

**Table 4:** Treatment effects on food choice and knowledge.

Measures	Actual food choice				Good food ID						
	T^a^ (n=27), mean (SD)	C^b^ (n=31), mean (SD)	Statistics			T (n=27), mean (SD)		C^a^ (n=31), mean (SD)		Statistics	
			*P* value^c^	*d* ^d^					*P* value		*d*
Number of good foods chosen/identified in 2 days (0 to 4 for choice; 0 to 8 for ID; day 1 + day 2)^e^	2.48 (1.19)	1.10 (1.08)	<.001	1.25	7.30 (1.64)		6.94 (1.31)		.048		0.25
Change in the number of good foods chosen/identified (–2 to 2 for choice; –8 to 8 for ID; day 2 – day 1)^e^	–0.11 (–0.93)	–0.13 (1.02)	.92	0.02	–0.26 (0.59)		0.23 (1.12)		.15		–0.54

^a^T: treatment.

^b^C: control.

^c^*P* values are reported from Mann-Whitney tests.

^d^d: Cohen *d*.

^e^Only the responses available on both days for schools A and B were used for comparison.

### Objective 2

**Figure 4:**
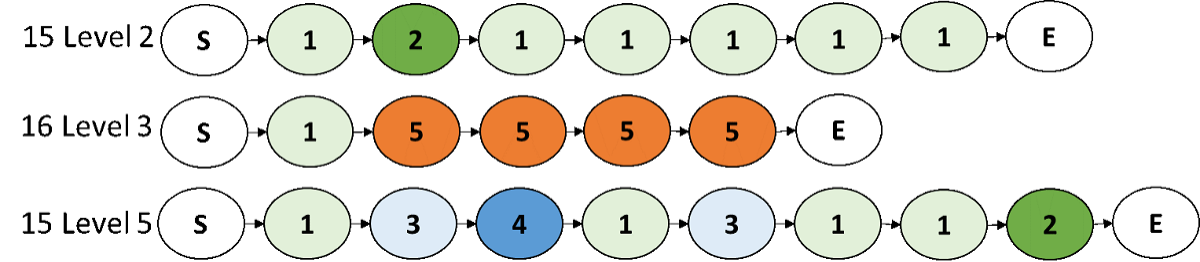
Sample sequences.

A total of 842 sequences were collated from the approximately 65,000 game play actions/clicks. Out of these, 115 sequences from repeated plays of random game levels by 38 children were removed, resulting in 727 first-encounter sequences for analysis. The telemetry data analysis indicated high variability in game play patterns among the children (Table 5). For example, children played 15 levels in 2 sessions, on average, ranging from a low of only 2 levels to a high of 23 levels. Coefficient of variation indicated that the largest variance among the variables was in the proportion of scoring activities that were highly rewarded (Sum2_Sum2+4), with an average of 0.17, ranging from 0.003 to 0.98. The descriptive summary in Table 6 shows that there was also a large variation in feature use among children and types of features. The variation was illustrated by the sample sequences shown in Figure 4. While child 15, in Figure 4, did not perhaps know that they could throw bad foods at level 2, they learned to use all features by level 5. In contrast, child 16 was only hit by the robots and could not find a way to fight back. Across different players and their levels of play, the number, type, and sequences of states greatly varied and was reflective of their game experience.

**Table 5:** Summary statistics of game play measures.

MDA^a^ components and game play measures		Min^b^		Mean		Max^c^		Variance		Coefficient of variation
**Game mechanics**										
	Level_max	2.00	14.78		23.00		21.73		31.54	
	Avg_Seqlen	8.96	65.77		145.42		893.28		45.44	
**Game dynamics**										
	Avg_transition	3.64	22.51		40.37		76.70		38.91	
**Game strategies**										
	Sum2_Sum2+4	0.003	0.17		0.98		0.05		131.53	
**Food learning**										
	AvgGFact	0.00	0.43		1.69		0.18		98.67	
	AvgBFact	0.00	0.12		0.60		0.02		117.85	

^a^MDA: mechanics, dynamics, aesthetics.

^b^Min: minimum.

^c^Max: maximum.

**Table 6:** Frequency by state for selected levels.

State	Level 1 (n=50)^a^			Level 10 (n=41)			Level 20 (n=8)^b^		
	Min^c^	Max^d^	Mean	Min	Max	Mean	Min	Max	Mean
1	0	5	2.16	0	6	2.70	2	3	2.63
2	0	57	2.02	0	106	2.98	0	19	2.88
3	0	136	32.94	0	175	61.68	0	116	67.25
4	0	17	5.64	0	22	9.15	0	21	8.75
5	0	14	1.76	0	10	4.27	0	11	6.00

^a^Two children assigned to the treatment group were absent for both exposures.

^b^This is the last level with number of players n>5.

^c^Min: minimum.

^d^Max: maximum.

**Figure 5:**
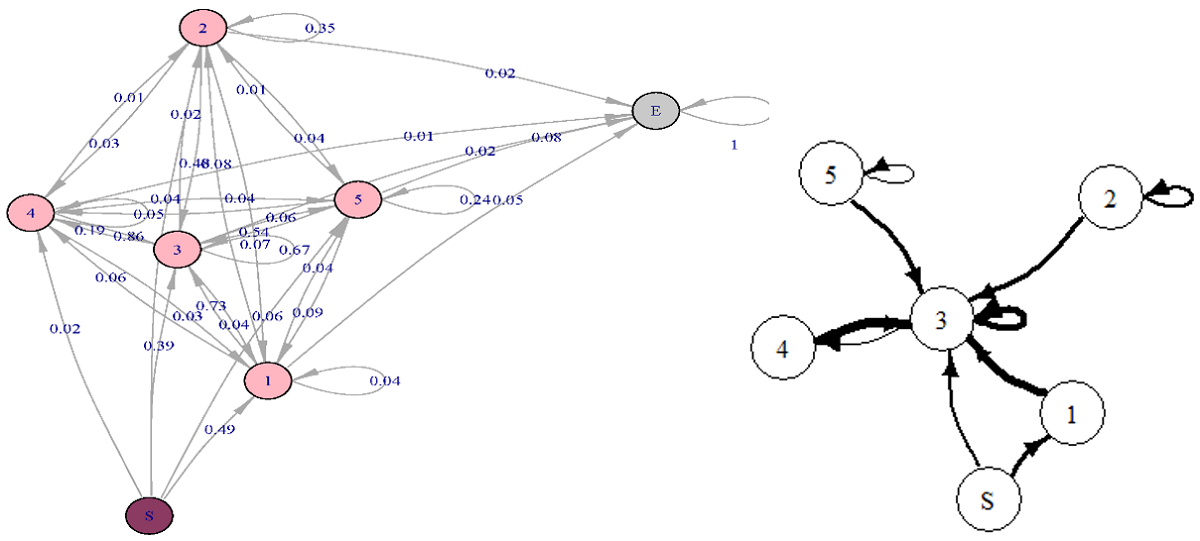
Markov chain for all children who played level 1 (n=50): (left) detailed plot with all transitions and (right) simplified plot with transition probabilities >0.1. The thickness of the lines is proportional to the transition probability.

From the Markov chain model in Figure 5, we observe that overall, bad food shot (state 3) played a central role in the game. It could immediately follow all other activities but was mostly followed only by itself (P_33_=.67) or robots being destroyed by food shots (P_34_=.19). We also note that good food shield was seldom followed by destroy by shield (P_12_=.08), suggesting that children failed to identify and take advantage of this highly rewarded transition throughout the experiment. This finding also confirms the theory that children experience many different learning styles demonstrated by their varied game play patterns.

### Objective 3

Table 7 shows the correlation between the outcome (GoodChoice) and the game play measures. Only AvgBFact had a significant association with GoodChoice (Kendall τ=–.32, *P*=.04). However, both AvgBFact and AvgGFact were retained after the stepwise feature selection procedure. To explore the possible associations, both variables were included in the regression model. The results are shown in models 1 (assumed normal) and 3 (assumed Poisson) in Table 8. The normality assumption for model 1 is satisfied using the Shapiro-Wilk normality test.

**Table 7:** Kendall rank correlation coefficients and significance test results.

Measures	GoodChoice	*P* value
Level_max^a^	–0.11	0.48
Avg_Seqlen^b^	0.10	0.50
Avg_transition^c^	–0.01	0.95
Sum2_Sum2+4^d^	–0.08	0.58
AvgGFact^e^	0.17	0.25
AvgBFact^f^	–0.32	0.04

^a^Level_max: maximum level played.

^b^Avg_Seqlen: average sequence length across levels played.

^c^Avg_transition: average number of transitions to a different state in a level across all levels played.

^d^Sum2_Sum2+4: proportion of robots destroyed by shield.

^e^AvgGFact: average number of nutrition facts of good foods read in a level.

^f^AvgBFact: average number of nutrition facts of bad foods read in a level.

**Table 8:** Regression results for GoodChoice^a^ (n=25).

Measures	Assumed normal					Assumed Poisson			
	Model 1, coefficient (robust SE)	*P* value	Model 2, coefficient (robust SE)	*P* value	Model 3, coefficient (robust SE)		*P* value	Model 4, coefficient (robust SE)	*P* value
Intercept	2.89 (0.31)	<.001	2.44 (0.39)	<.001	1.06 (0.09)		<.001	0.86 (0.14)	<.001
AvgGFact^b^	1.12 (0.91)	0.23	1.15 (0.62)	0.08	0.44 (0.17)		0.01	0.49 (0.15)	0.001
AvgBFact^c^	–9.51 (3.33)	0.01	–8.38 (3.60)	0.03	–4.25 (1.53)		0.01	–3.81 (1.45)	0.01
GoodBase^d^	—^e^	—	0.92 (0.38)	0.03	—		—	0.36 (0.13)	0.01
Goodness of fit	Adjusted *R*^2^=.31	—	Adjusted *R*^2^=.47	—	AIC^f^: 83.55		—	AIC: 83.59	—

^a^Outcome variable: number of good foods chosen in day1+day2 (0-4). Only the data available on both days in schools A and B were used.

^b^AvgGFact: average number of nutrition facts of good foods read in a level.

^c^AvgBFact: average number of nutrition facts of bad foods read in a level.

^d^GoodBase: binary variable indicating whether the player’s baseline preference was healthy or not healthy.

^e^Not applicable.

^f^AIC: Akaike information criterion.

Consistent with Kendall rank test, models 1 and 3 agreed that reading more facts about bad foods is associated with worse food choices. Furthermore, we see that AvGFact may have a positive effect, as shown in model 3. Although the data were collected from a randomized experiment, it is still a concern that these relationships may be due to the children’s food preference at baseline. To control for their food preference at baseline, we included a binary variable GoodBase indicating whether the player’s baseline preference was healthy (coded as 1) or not healthy (coded as 0). This variable was obtained from an open-ended question in the pretest survey asking the children to write down their favorite food. These open-ended answers were then coded by two raters independently. Disagreements were resolved via a consensus meeting. If no consensus could be reached, a third rater made the final decision. Cohen kappa for overall raters’ agreement was 0.72 before consensus and 0.91 after consensus [58].

The results after including the baseline food preference are shown in models 2 (assumed normal) and 4 (assumed Poisson). Not surprisingly, baseline preference had a positive effect on actual food choice. However, the negative effect of AvgBFact still remained, and the potential positive effect of AvgGFact became evident.

## Discussion

### Principal Findings

Video games with implicit learning strategies, perceived by children as a fun activity and not a learning tool, present a great opportunity to change children’s health behaviors by delivering relevant knowledge implicitly. However, without explicit education, whether and how the implicit knowledge can be internalized and result in behavioral change is uncertain. Despite the null effect of some video games found in recent studies [9,12,14], our study provides new evidence by comparing fooya! with a standard board game not designed to deliver implicit knowledge on healthy eating. Our result showed that a mobile game that embeds implicit learning in the game mechanism can positively impact children’s actual food choices, consistent with the theory that the tacit knowledge learned from game play can be used to make good decisions [15] and that game play experience can translate to real-world food choices [35-37]. This result is also consistent with empirical studies that have examined the effect of serious video games on health behaviors [21,22].

Furthermore, our analysis of children’s play patterns showed significant variations in game play among participants, confirming theories that suggest different children can experience different learning styles [41-43,45]. Sharma and colleagues [7] also found a high variation in time spent in game play and levels played. To the best of our knowledge, our study presents the first attempt to find support for a wide range of learning patterns among players that are associated with different outcomes. Our results show that food choice was not influenced by levels played, same as reported by Sharma and colleagues [7]. Instead, it was influenced by the food facts read in the game: reading more facts about unhealthy foods was associated with more unhealthy food choices and vice versa. This finding is counterintuitive, as one would expect that reading facts about unhealthy foods would lead to decreased choice of unhealthy foods. While our game did not suggest whether the food was good or bad, one possible explanation is that the children were not influenced by the content of the food facts but the impression (the number of views) of the food fact. This is in line with the phenomenon found in studies investigating the effects of advergames [59], in which food choice was affected by the foods advertised in the game. This may be a result of priming [60], which posits that a person’s response to the current stimuli (actual food choice) can be influenced by previous stimuli (facts read). Despite the negative effect of bad food facts, our subjects looked up more good food facts than bad food facts, which was one of the drivers of the overall positive effect of the game.

Our results provide several implications for game design. First, serious health games with implicit education strategies as the primary mechanism can be an effective intervention for improving healthy eating behaviors. Players are able to enjoy the game without noticing that they are being educated. Second, video game designers may want to limit the display of unhealthy foods in the game, especially games that present players with nutritional facts on foods [9], unless some form of protective or explanatory messages are also in place. Furthermore, game designers may also want to insert displays of food facts within the level to make it easier for players to associate the food facts with the change in the avatar’s body shape and speed. Third, video games may be coupled with other interventions, whether in-game or not, for children with different game play patterns such as those who read many bad food facts in the game. This personalization of the game or intervention is made possible by associating the actual food choice with game play behaviors measured and quantified using detailed game telemetry.

### Limitations and Future Research

This research provides several directions for future extensions by reviewing its limitations. The major limitation of this study is the small sample size. The authors recruited 104 students, but due to the potential bias in the behavioral outcome in one school, data from only 58 students were analyzed for objectives 1 and 3. Due to the observed large effect size, the small sample size is not a problem for objective 1. However, for objective 3, the small sample size prevents us from including the full set of game play measures and controls such as gender and other demographics. It also limits the reliability of regression estimates, especially for the 3-variable models. This small sample size, short game sessions with fixed duration, and the homogeneous subject groups also limit the generalizability of our results. However, these limitations are not unique to this paper, as can be seen in recent game-related literature. For example, existing literature reporting studies with sample size of 46 [61], 20-minute game exposure time [62], or subjects recruited from a single institution [61,63] still provide novel and useful insights. Second, the fact that the actual food choices were made immediately after playing the game limits our observed effect to be the immediate effect. Future longitudinal studies could impose a delay before making the actual food choice to minimize the priming effect and examine whether the game effect is sustained over a longer period and how long it takes for the effect to be internalized.

Third, the kids were not required to consume the chosen food items on site, raising a concern that the strategically thinking kids may choose good foods just to leave a good impression. This concern may be minimal, as the students were not told the purpose of the study. Their choices were also not observed by the people whose opinions they care about, such as their classmates or teacher. With ample sample size and data, future longitudinal studies may further minimize this concern by controlling for students’ academic performance, which can be used as a proxy for smartness. Fourth, the choice of a board game (Uno) as a control only allows us to compare the effect of fooya! with a nonserious game. With established effects of games with implicit education strategies, future studies may further examine whether fooya! is preferred to other modes of education on healthy nutrition. Fifth, the BMI in our control group is slightly higher than that in our treatment group, raising a concern that we may have overestimated the size of our main effect. However, the difference in BMI is not statistically significant between the two groups. Last, we use the game play pattern of the first play as a proxy for learning styles.

Future studies may establish the association between learning styles and game play patterns such that tailoring of the intervention can happen before game play. With the overall positive effect found in this study, future studies may also carefully craft and compare the effect of individual implicit education strategies with the results of explicit education components. In addition, analyzing the game play patterns of repeated plays of a level to understand players’ established routines rather than first-encounter effects is another avenue for exploration. With more high-quality evidence obtained from longitudinal studies that address these limitations, there is potential for such digital interventions to act as a digital vaccine for pediatric obesity.

### Conclusions

Implicit and gamified learning about healthy eating delivered via a mobile app can significantly improve children’s food choices immediately after the game. While additional scientific evidence is needed to confirm that such apps can serve as a digital vaccine with long-term impact, this study provides novel insights about the potential drivers of the observed positive short-term effect. We measured and quantified the variation in children’s game play patterns that may be associated with children’s health behavior outcomes. Specifically, the positive main effect may be strengthened when players read more nutrition facts about healthy foods in the game. However, the effect is compromised when the players read more facts about unhealthy foods. Results of this study provide promising directions for the use of a viable alternative to improve children’s eating habits and help address the pediatric overweight and obesity epidemic in the long term. Future research with large scale, longitudinal RCTs is needed to fully understand how game play patterns with their underlying learning styles may facilitate desired behavioral outcomes.

## Appendix

Multimedia Appendix 1 CONSORT-eHEALTH checklist (V. 1. 6. 1).

## Abbreviations

Avg_Seqlen: average sequence length across levels played
Avg_transition: average number of transitions to a different state in a level across all levels played
AvgBFact: average number of bad food facts read per level
AvgGFact: average number of good food facts read per level
GoodBase: binary variable indicating whether the player’s baseline preference was healthy or not healthy
GoodChoice: total number of good foods chosen in the 2 sessions
Level_max: highest level played in the 2 sessions
MDA: mechanics, dynamics, aesthetics
RCT: randomized controlled trial
Sum2_Sum2+4: proportion of robots destroyed by shield
